# Quaternary Ammonium-Bearing Perfluorinated Polymers for Anion Exchange Membrane Applications

**DOI:** 10.3390/membranes10110306

**Published:** 2020-10-26

**Authors:** Seunghyun Lee, Hyejin Lee, Tae-Hyun Yang, Byungchan Bae, Nguyen Anh Thu Tran, Younghyun Cho, Namgee Jung, Dongwon Shin

**Affiliations:** 1 Fuel Cell Laboratory, Korea Institute of Energy Research, Daejoen 34129, Korea; seunghyun1502@kier.re.kr (S.L.); hyejinlee@kier.re.kr (H.L.); thyang@kier.re.kr (T.-H.Y.); bcbae@kier.re.kr (B.B.); 2 Graduate School of Energy Science and Technology, Chungnam National University, Daejeon 35015, Korea; 3 Department of Renewable Energy Engineering, University of Science and Technology, Daejeon 34113, Korea; 4 Department of Energy Systems Engineering, Soonchunhyang University, Asan 31538, Korea; tnathu76@gmail.com (N.A.T.T.); yhcho@sch.ac.kr (Y.C.)

**Keywords:** perfluorinated polymer, anion exchange membrane, morphology, ion conductivity, flow-electrode capacity deionization

## Abstract

Perfluorinated polymers are widely used in polymer electrolyte membranes because of their excellent ion conductivity, which are attributed to the well-defined morphologies resulting from their extremely hydrophobic main-chains and flexible hydrophilic side-chains. Perfluorinated polymers containing quaternary ammonium groups were prepared from Nafion- and Aquivion-based sulfonyl fluoride precursors by the Menshutkin reaction to give anion exchange membranes. Perfluorinated polymers tend to exhibit poor solubility in organic solvents; however, clear polymer dispersions and transparent membranes were successfully prepared using *N*-methyl-2-pyrrolidone at high temperatures and pressures. Both perfluorinated polymer-based membranes exhibited distinct hydrophilic-hydrophobic phase-separated morphologies, resulting in high ion conductivity despite their low ion exchange capacities and limited water uptake properties. Moreover, it was found that the capacitive deionization performances and stabilities of the perfluorinated polymer membranes were superior to those of the commercial Fumatech membrane.

## 1. Introduction

Ion exchange membranes (IEMs) play an important role in electrochemical and separation applications, such as fuel cells, flow batteries, electrolysis, and electro-deionization [1,2,3,4]. IEMs can be categorized into cation exchange membranes (CEMs) and anion exchange membranes (AEMs) according to the specifications of the transport ions [5]. Both CEMs and AEMs require excellent ion conductivity and long-term stability, regardless of their application. Although there are many commercially available CEMs, such as Nafion^®^, Aquivion^®^, NEOSEPTA^®^, and Fumapem^®^, very few AEMs with sufficient ion conductivity and long-term durability have been commercialized. Intrinsically, the anion (e.g., OH^−^) mobility of such a membrane is significantly lower than the cation (e.g., H^+^) mobility [6]. Moreover, AEMs require excellent chemical stability because organic materials are generally unstable under alkaline conditions [7,8,9]. Composites with inorganic nano-particles can further improve the chemical stability and electrochemical performance; however, the intrinsic properties of the polymer matrix are most important to AEM performance [10,11,12]. Therefore, advanced AEMs are receiving increasing attention for their use in various applications.

Significant research was recently conducted to develop advanced AEMs, whereby the ion conductivity and long-term reliability have received particular attention [13,14,15]. Many research groups have reported hydrocarbon-based aromatic polymers, such as polysulfones, polyoxidazoles, polyimides, polyketones, and polyphenylenes, due to their cheap and easy synthetic routes [16,17,18,19,20,21]. In addition, their good thermal stabilities and mechanical strengths can expand their possible fields of application. Among the various kinds of aromatic polymer backbones reported to date, polyphenylene is the state of art in AEMs owing to its excellent chemical stability [22]. Nevertheless, its low ion conductivity remains an issue, as the rigid C‒C bond reduces water uptake. Although the low ionic conductivities of aromatic polymers can be enhanced by the syntheses of multi-block copolymers, the preparation of multi-block copolymers with polyphenylene structures is challenging because of the lower reactivity and poor solubility of this structure [23].

Perfluorinated ionomers have been recognized for their outstanding ion conductivities and chemical stabilities in their application as CEMs, although no such analog exists for AEMs [24,25,26,27,28,29]. Perfluorinated ionomers, such as Nafion^®^ and Aquivion^®^, are composed of a flexible tetrafluoroethane-based main chain and fluoroalkyl side chains. It is well known that the extremely hydrophobic main chain and hydrophilic ionic groups in the flexible fluoroalkyl side chains result in distinguishable hydrophilic-hydrophobic phase separation, which facilitates fast ion transport [30,31,32]. In addition, their well-defined morphologies assist water transport and they tend to feature reduced water absorption because of the highly hydrophobic nature of the perfluorinated chemical structure [33,34,35].

Based on these properties, perfluorinated polymers are good candidates for AEM applications. The physical properties of perfluorinated polymers depend on their chemical structure, such as the length of the side-chain, the number of repeating units, and the equivalent weight (EW). Although the syntheses of perfluorinated polymer-based AEMs is challenging owing to their poor solubilities in organic solvents and safety issues, a few studies have been conducted [36,37,38]. These perfluorinated ionomers were synthesized from sulfonyl fluoride precursors followed by quaternization via the Menshutkin reaction. In addition, various cationic groups, such as ammonium, guanidinium, and piperazinium, have been introduced into perfluorinated polymer precursors (e.g., Nafion, 3M, and Huaxia Shenzhou New Materials Company Ltd., Zibo City, Shandong, China) [39,40,41,42,43]. However, Aquivion-based AEMs have not been studied.

Thus, we herein report the quaternization of Nafion- and Aquivion-based perfluorinated precursors with trimethylamine to prepare perfluorinated ionomers. The effect of the perfluorinated chemical structure and equivalent weight on the AEM properties are then compared with those of a commercial hydrocarbon-based Fumatech ionomer. Subsequently, the water uptake, swelling ratio, ion conductivity, flow electrode capacitive deionization (FCDI) performance, and stability are tested to demonstrate the feasibility of using perfluorinated polymers for AEM applications.

## 2. Materials and Methods

### 2.1. Materials

The Nafion^®^ sulfonyl fluoride precursor (Nafion-SO_2_F, EW = 1100 g/mol) was obtained from Alfa Aesar (Ward Hill, MA, USA). The Aquivion^®^ sulfonyl fluoride precursor (Aquivion-SO_2_F, EW = 830 g/mol), *N*,*N*-dimethylformamide (DMF, anhydrous, ≥99.8%), *N*-methyl-2-pyrrolidone (NMP, anhydrous, ≥99.9%),) trimethylamine (TMA, 25 wt% in H_2_O) solution, sodium hydroxide (NaOH, pellets, ≥95%), sodium chloride (NaCl, pellets, ≥99%), hydrochloric acid (HCl, ACS reagent, 37%) solution, silver nitrate (AgNO_3_, ACS reagent, ≥99.9%), sodium nitrate (NaNO_3_, ACS reagent, ≥ 99.0%), and Nafion^®^ membranes were purchased from Sigma Aldrich (St. Louis, MO, USA) and used as received. The activated carbon materials (MSP-20X with a mean diameter of 9 µm) were purchased from Kansai Coke & Chemicals (Hyogo, Japan). The FAA-3-50 membrane was supplied by Fumatech (St. Ingbert, Germany) as a reference.

### 2.2. Quaternization and Membrane Fabrication

**Scheme 1:**
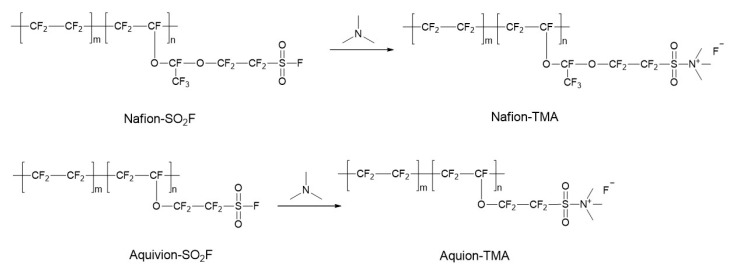
Quaternization of the Nafion- and Aquivion-based perfluorinated polymer precursors.

Introduction of the quaternary ammonium group into the Nafion-SO_2_F and Aquivion-SO_2_F precursors were conducted via the Menshutkin reaction with TMA at 220 °C for 9 h, as outlined in Scheme 1. The reaction mixture was heated and pressurized using an autoclave under a nitrogen atmosphere. The quaternized polymers (Nafion-TMA and Aquivion-TMA) were purified by washing several times with deionized water to remove any residual DMF solvent and TMA, then dried at 80 °C under vacuum. The resulting polymer was re-dissolved in a 2 w/v% solution of NMP at 120 °C for membrane fabrication. The polymer solution was cast onto a flat glass plate after filtering through a 5 μm syringe filter, and then dried at 80 °C under vacuum. The membrane thickness was controlled at 50 μm to eliminate the effect of thickness on the membrane properties. To convert the counter ion of the quaternary ammonium cation to the Cl^−^ form, the membrane was immersed in a 3 M NaCl solution for 24 h and then rinsed with deionized water.

### 2.3. Characterization

Fourier transform infrared (FT-IR) spectra were obtained to examine changes in the chemical structures of the perfluorinated polymers after quaternization and degradation. For this purpose, a Nicolet-5700 FT-IR spectrometer (Thermo Electron Corporation, USA) equipped with an attenuated total reflectance (ATR) accessory was employed. Spectra were recorded over a wavelength range of 4000–650 cm^−1^ with a scan wavenumber of 4 cm^−1^.

The anion conductivities of Nafion-TMA and Aquivion-TMA were measured by electrochemical impedance spectroscopy using a Solartron 1260 impedance/gain-phase analyzer and a Solartron 1287 electrochemical interface with a WonATech 4-probe conductivity cell. The temperature and humidity were regulated by an electrochemical performance test station (CNL). The impedance resistance (R) was obtained from the x-intercept of the Nyquist plot in the frequency range of 10^−1^ to 10^5^ Hz, and the ion conductivity (*σ*) was calculated using Equation (1):

(1) $$\sigma =\;\frac{D}{W\times T\times R}$$

where *D* is the distance between the electrodes, and *W* and *T* are the width and thickness of the sample, respectively.

The water uptake of the membranes was calculated based on the weight difference between the dry and wet membranes. The weight of the dry membranes was obtained after drying at 120 °C under vacuum for 24 h. The membrane was then immersed in deionized water at room temperature for 24 h to measure the weight of the wet membrane.

The swelling ratio of the membrane was determined from the difference between the wet and dry dimensions. The wet dimension was measured after soaking in deionized water for 24 h and the dry dimension was measured after drying the wet samples at 120 °C for 24 h under vacuum. The area swelling ratio (*A_s_*) was calculated using Equation (2):

(2) $$A_{s}=\left(\frac{A_{w}-A_{d}}{A_{d}}\right)\times 100$$

where *A_w_* and *A_d_* are the areas of the wet and dry membranes, respectively.

The thickness swelling ratio was obtained following the same method as the area swelling ratio.

The characterization factor (CF) of the membrane was obtained from the ratio of ion conductivity to water uptake. The ion exchange capacity (IEC) of the membrane was measured by the Mohr titration method [44]. To convert the counter ion into Cl^−^, the 2 × 2 cm^2^-sized membrane samples were immersed in a 0.5 M HCl solution for 24 h, and then immersed in a 0.2 M NaNO_3_ solution (20 mL) for 24 h. The NaNO_3_ solutions were then titrated with a 0.01 M AgNO_3_ ($C_{{\mathrm{AgNO}}_{3}})$ standard solution using K_2_CrO_4_ as a colorimetric indicator. The membranes in the solution were dried at 120 °C under vacuum for 24 h and weighed. The IEC was calculated using the amount of AgNO_3_ ($\Delta V_{{\mathrm{AgNO}}_{3}}$) used for titration and the weight of the dried membrane (*W_d_*). The IEC value was calculated using Equation (3):

(3) $$\mathrm{IEC}=\frac{\Delta V_{{\mathrm{AgNO}}_{3}}\times C_{{\mathrm{AgNO}}_{3}}}{W_{d}}$$

### 2.4. Transmission Electron Microscopy (TEM)

Transmission electron microscopy (TEM) observations were performed on a JEOL JEM-2100 F with an accelerating voltage of 200 kV. The ionic group was stained with phosphotungstic acid. The membranes were sectioned to a thickness of 90 nm with an ultramicrotome after being embedded in epoxy resin and placed on copper grids.

### 2.5. Alkaline Stability Tests

The chemical stabilities of the AEMs under alkaline conditions (0.5 and 1 M NaOH) were estimated by monitoring changes in their IEC values and FT-IR spectra. The membranes were immersed in an alkaline solution, and samples were taken for analysis every 24 h and washed with deionized water to remove the residual alkaline solution. The entire process was conducted under an argon atmosphere to avoid poisoning by carbon dioxide. The IEC values were determined and FT-IR spectral measurements were carried out following the procedures described above.

### 2.6. Flow-Electrode Capacitive Deionization (FCDI) Desalination

For the flow-electrode capacitive deionization desalination tests, an electrode suspension was prepared by adding 20 wt% activated carbon to deionized water containing 2.5 wt% sodium chloride. The FCDI cell consisted of a pair of graphite current collectors with a carved flow path (1 mm wide, 1 mm deep) and polycarbonate substrates. The anionic and cationic exchange membranes were located on the current collector. The flow electrode slurry was pumped into the anode and cathode channels separately and drained from the FCDI cell using a peristaltic pump. The saline feed stream (35 g/L NaCl solution) was also pumped in and out from the center channel of the FCDI cell independently. Desalination was carried out by applying a constant cell potential difference of 1.2 V [45,46].

## 3. Results

### 3.1. Syntheses of the Perfluorinated Ionomers

**Figure 1:**
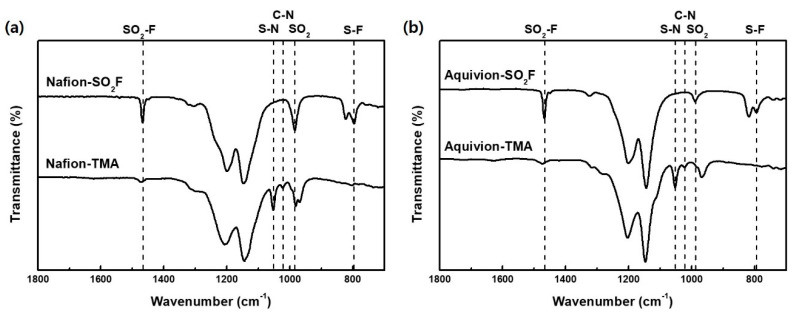
FT-IR spectra of the (**a**) Nafion- and (**b**) Aquivion-based polymers before and after quaternization.

Nafion-TMA and Aquivion-TMA were synthesized from their sulfonyl fluoride precursors via the Menshutkin reaction with TMA, as outlined in Scheme 1. The reaction was carried out using an autoclave reactor at 220 °C and 10 bar pressure due to the low solubility of the perfluorinated polymers. The changes in the chemical structures of the perfluorinated polymers after quaternization were confirmed by FT-IR spectroscopy. The intense peak at 1467 cm^−1^ was assigned to an asymmetric stretch of the SO_2_-F, and the peaks at 823 and 796 cm^−1^ were associated with the stretching vibration of the S–F group. Peaks related to the sulfonyl fluoride end group disappeared after quaternization, and a new peak corresponding to the C–N vibration of the quaternary ammonium group was observed at 1020 cm^−1^, in addition to a peak attributed to the S–N stretching vibration at 1051 cm^−1^, as shown in Figure 1. Furthermore, the stretching peak of O=S=O was slightly shifted, which further confirmed the successful quaternization.

### 3.2. Solubilities of the Perfluorinated Ionomers and Membrane Preparation

**Figure 2:**
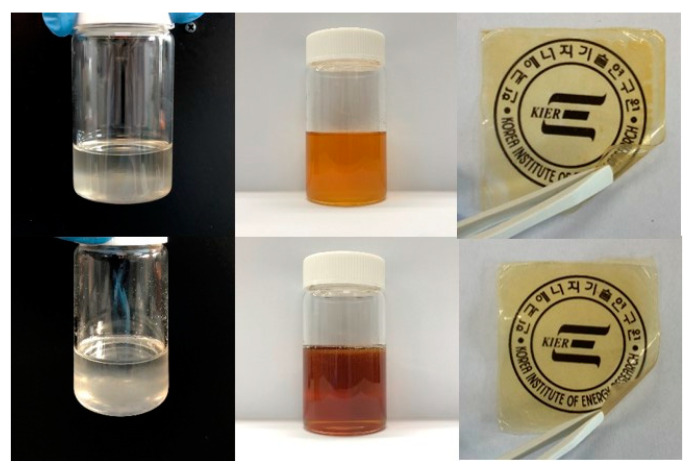
Photographic images of the Nafion-TMA (trimethyl amine, upper) and Aquivion-TMA (lower)-based dispersions in *N*,*N*-dimethylformamide (DMF, **left**) and *N*-methyl-2-pyrrolidone (NMP, **middle**), and the resulting membranes (**right**).

DMF was used as the solvent for the quaternization process because it is the best solvent for perfluorinated precursors [47,48]. However, as shown in Figure 2, the quaternized perfluorinated ionomers were not completely soluble in the DMF solvent, despite exhibiting a relatively good solubility in NMP. Although the preparation of a 100% homogeneous polymer solution was challenging, even in NMP, a transparent precipitate-free dispersion was prepared for membrane casting. Moreover, transparent and ductile membranes were prepared after filtering the solution through a 5.0 µm syringe filter. The counter-anion of the TMA cation was converted to a chloride anion to measure the physical properties of the ionomer with the same ionic form.

### 3.3. Physical Properties of the Quaternized Polymers

The IEC value represents the amount of ion-conducting functional groups relative to the mass of the polymer. Therefore, some key physical properties of polymer electrolyte membranes, such as the water uptake, swelling ratio, and ion conductivity, depend on their IEC values. Additionally, the degree of functionalization can be estimated by measuring the IEC values. The measured IEC values of the Nafion-TMA (0.88 meq/g) and Aquivion-TMA (1.06 meq/g) membranes were consistent with the empirically expected IEC values (i.e., 0.91 and 1.15 meq/g), indicating successful quaternization of the perfluorinated polymers.

The perfluorinated ionomers were found to exhibit lower water uptakes and swelling ratios than the Fumatech ionomer because of their lower IEC values, as outlined in Table 1. In addition, the superhydrophobic nature of the perfluorinated chemical structure reduces the water uptake and swelling ratio despite the presence of a flexible aliphatic polymer backbone and side chains, which contrasts with the hydrocarbon-based aromatic nature of the Fumatech ionomer polymer. It should be noted here that water uptake is an important property in terms of determining the ion transport behavior because water molecules play a role as an additional ion conductor. This resulted in the perfluorinated polymers exhibiting significantly higher ion conductivities than the Fumatech ionomer. However, a high-water uptake induces excessive swelling of the membrane, resulting in mechanical failure. Therefore, the real excellence of the polymer electrolyte can be evaluated by a characteristic factor, which is the ratio of the ion conductivity to the water volume fraction. When the perfluorinated polymers were compared with the Fumatech ionomer in terms of their ion conductivities, the values corresponding to the perfluorinated ionomers were almost three to four times superior to those of the Fumatech ionomer. On the other hand, the perfluorinated ionomers presented characteristic factors that were approximately four times greater than that of the Fumatech ionomer. Moreover, the Aquivion-TMA membrane exhibited a higher characteristic factor than the Nafion-TMA membrane. This was attributed to the short side-chain of the Aquivion polymer, which reduced the water uptake and swelling ratio, in addition to the high IEC that resulted in an improved ion conductivity. The higher crystallinity imparted by the short side-chain appears to play a role in suppressing the water uptake.

**Figure 3:**
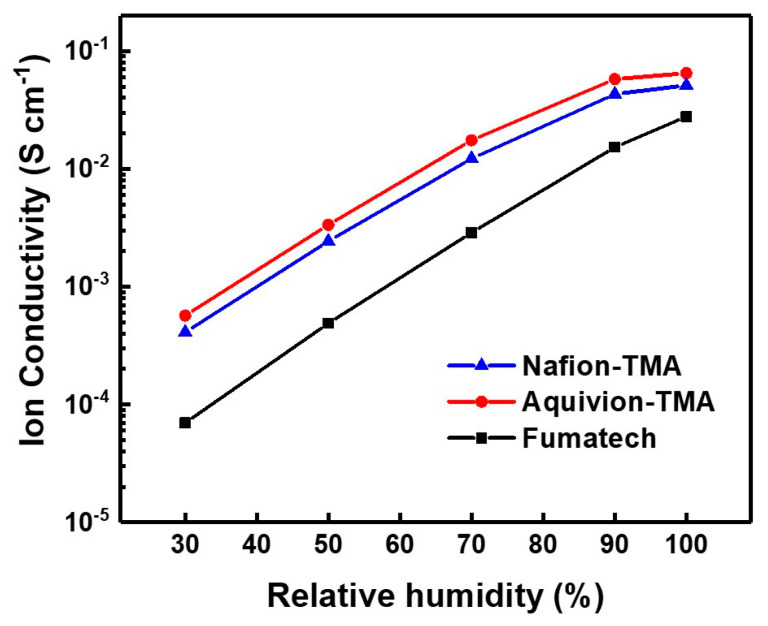
Ion conductivities of the Nafion-TMA, Aquivion-TMA, and Fumatech membranes at different relative humidities.

The RH dependence of the chloride anion conductivities of the perfluorinated ionomers were then measured, and the results were compared to those of the Fumatech ionomer at 70 °C under different RH environments. Despite the low IEC values of the perfluorinated ionomers, their chloride anion conductivities outperformed that of the Fumatech ionomer in all RH ranges, as shown in Figure 3. In addition, the ion conductivities of the perfluorinated ionomers were less dependent on the RH than that of the Fumatech ionomer, which is evidence for the formation of an effective ion-conducting pathway. Furthermore, the Aquivion-TMA membrane exhibited a slightly higher chloride anion conductivity than the Nafion-TMA membrane because of the differences in their IEC values. Consequently, Aquivion-TMA showed the highest ion conductivity, which was approximately 10 times higher than that of Fumatech at 30% RH.

**Figure 4:**
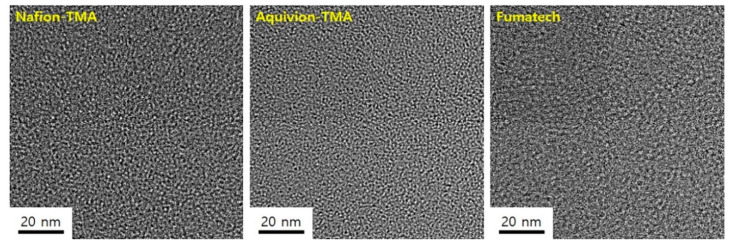
TEM images of the Nafion-TMA, Aquivion-TMA, and Fumatech membranes.

Formation of the ion-conducting pathway was confirmed by TEM observations, as shown in Figure 4. All membranes exhibited a hydrophilic (dark region) and a hydrophobic (bright region) phase-separated morphology. Although the perfluorinated membranes exhibited narrow hydrophilic domains due to their flexible aliphatic chemical structures and low IEC values, the connectivity was superior to that observed in the hydrophilic domains of the Fumatech membrane. In addition, the perfluorinated polymers showed more distinct phase separation than the Fumatech membrane, because the extremely hydrophobic nature of the perfluorinated structure results in a clear contrast between the hydrophilic and hydrophobic domains. Considering the large difference between the IEC values of the Fumatech and fluorinated membranes, the morphological observations seem reasonable. Indeed, these morphology images support our conclusion that the perfluorinated polymers exhibit a high ion conductivity despite their low IEC values and water uptake properties.

### 3.4. Flow Electrode Capacitive Deionization Performance and Stability

**Figure 5:**
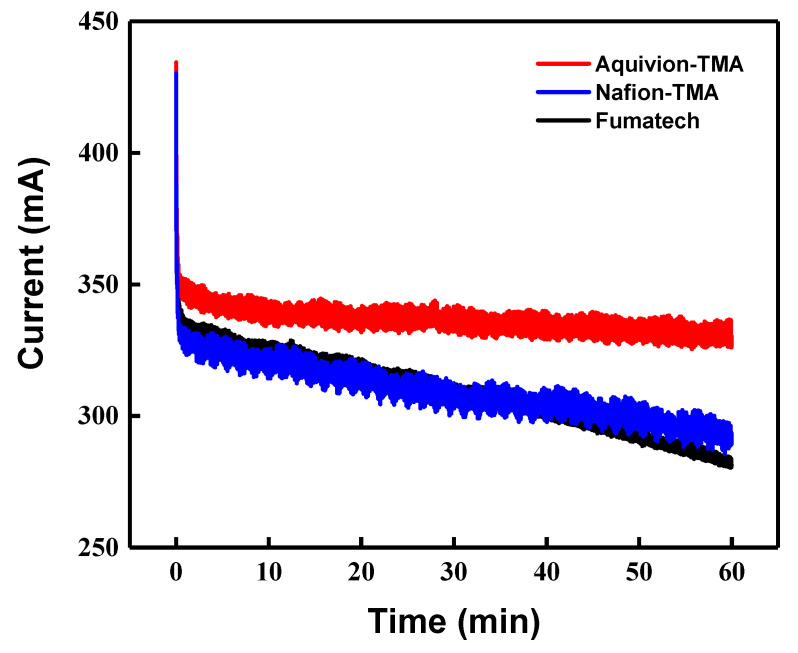
Variation in current during flow electrode capacitive deionization (FCDI) desalination using the different anion exchange membranes.

Desalination using a flow electrode capacitive deionization cell was performed to confirm the ion-exchange properties of the various anion exchange membranes, as shown in Figure 5. For FCDI operation, when the potential is applied to the FCDI cell, charged sodium and chloride ions in the feed stream migrate through the ion exchange membranes and are adsorbed onto the electric double layer of the carbon electrode by electrostatic interactions. Thus, the ions adsorbed onto the electrodes were drained, and a fresh electrode was continuously supplied into the FCDI cell. Therefore, sodium and chloride ions were continuously removed without the need for a discharge process, which is a distinct advantage of the FCDI desalination system over the conventional fixed-electrode CDI desalination approach. The measured current at a constant applied potential of 1.2 V therefore indicates the extent of salt removal during desalination. Nafion was used as a cation exchange membrane with various counter anionic exchange membranes. The constant measured current of ≈345 mA for the FCDI cell using Aquivion-TMA as an anion exchange membrane indicated that the salt ions were continuously removed during the operation time with a constant salt removal efficiency. On the other hand, for the Nafion-TMA and Fumatech membranes, the measured current (≈330 mA) during the initial FCDI operation was lower than that of the Aquivion-TMA membrane. Furthermore, the current gradually decreased as the operation proceeded. After 60 min, ≈12% of the initial current had been lost, which indicates that the ion separation and transportation performance of the Nafion-TMA and Fumatech membranes were continuously reduced under the desalination experimental conditions. These results confirmed that the Aquivion-TMA membrane demonstrated a much more stable and higher salt removal performance when utilized for FCDI desalination. This is due to the short side-chains present in the Aquivion membrane, which enhance the crystallinity and stability, as also discussed in the context of the CF value [49,50].

### 3.5. Alkaline Stabilities of the Quaternized Polymers Under a NaOH Atmosphere

**Scheme 2:**
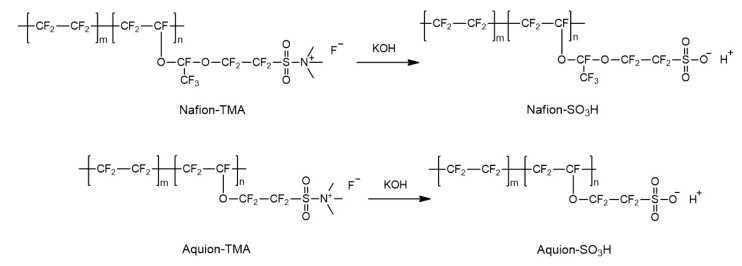
Possible degradation route of the perfluorinated ionomers via an S_N_2 nucleophilic displacement reaction.

**Figure 6:**
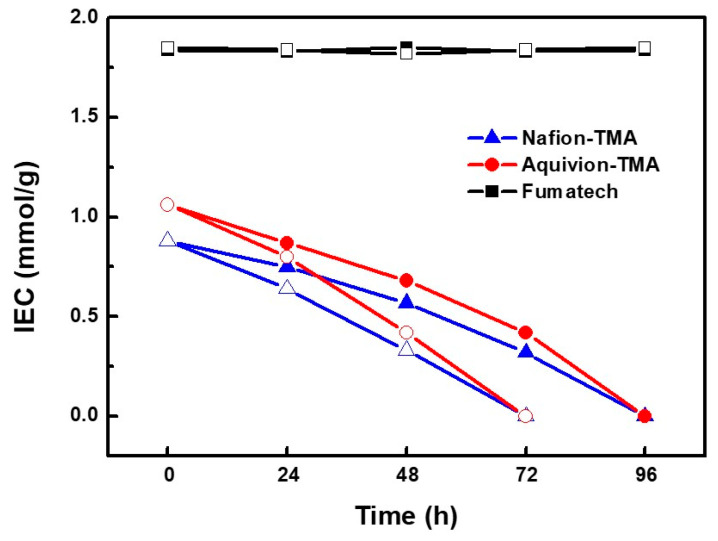
Alkaline stabilities of the Nafion-TMA, Aquivion-TMA, and Fumatech membranes in 0.5 (solid) and 1.0 M (open) NaOH solutions at 25 °C.

Scheme 2 presents the degradation pathway of the perfluorinated ionomers under strongly alkaline conditions, under which the electron-withdrawing characteristics of the perfluorinated chemical structure render it susceptible to nucleophile attack. Furthermore, the quaternary ammonium cationic group also interacts strongly with the hydroxide anion, and so the sulfonyl ammonium group can be hydrolyzed to give a sulfonic acid group. For these reasons, the alkaline stabilities of the perfluorinated ionomers were estimated by observing changes in their IEC values using 0.5 and 1.0 M NaOH solutions at room temperature. Figure 6 shows the reduction in the IEC values of the perfluorinated ionomers and the Fumatech ionomer as a function of time. As shown, the IEC values of both the Aquivion-TMA and Nafion-TMA membranes continuously decreased over time, and the perfluorinated ionomers lost almost all ionic groups after 72 and 96 h in 0.5 and 1.0 M NaOH solutions, respectively. However, the IEC value of the Fumatech ionomer was maintained because of its hydrocarbon-based chemical structure, which interacts only weakly with nucleophiles.

**Figure 7:**
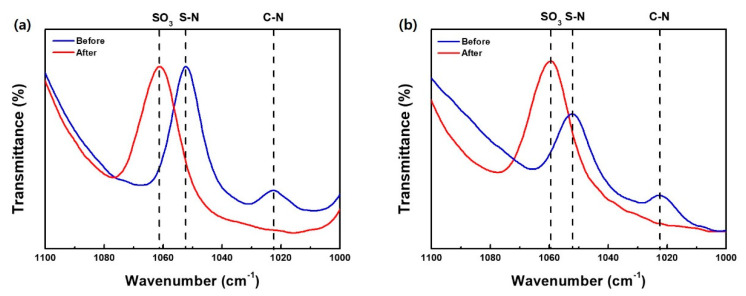
FT-IR spectra of the (**a**) Nafion-TMA and (**b**) Aquivion-TMA membranes before and after the alkaline stability test.

Changes in the chemical structures after the alkaline stability test were confirmed by FT-IR measurements (Figure 7), which indicated that hydrolysis of the quaternary ammonium groups was successful in the perfluorinated ionomers to yield sulfonic acid groups [51,52,53]. The symmetric SO_2_ stretching of the SO_3_^−^ group appeared at 1060 cm^−1^, while a band corresponding to C–N vibrations was observed at 1020 cm^−1^, and the peak at 1051 cm^−1^ originating from the stretching vibration of the S–N moiety in the sulfonyl ammonium group disappeared. This weak chemical stability exhibited by the quaternary ammonium groups can be enhanced by incorporating other stable functional groups, such as cyclic cations [29,30]. In addition, the introduction of a spacer group between the perfluorinated polymer chain and the functional group can improve the chemical stabilities of perfluorinated polymer-based AEMs. Nevertheless, the Nafion- and Aquivion-TMA membranes are suitable for application to a wide range of electrochemical systems that do not involve the use of strongly alkaline conditions. We will also report the enhanced stability of the perfluorinated ionomer through the use of various chemical approaches.

## 4. Conclusions

Perfluorinated ionomers were successfully synthesized from Nafion- and Aquivion-based precursors by quaternization with trimethylamine. To eliminate the effects of counterions on the membrane properties, chlorinated membrane forms were used for all physical property analyses. Both the Nafion-TMA (TMA: trimethylamine) and Aquivion-TMA membranes exhibited lower water uptakes and higher dimensional stabilities than commercial Fumatech ionomers due to their low ion exchange capacity (IEC) values and the extremely hydrophobic nature of their perfluorinated chemical structures. In addition, the perfluorinated ionomers showed outstanding ion conductivity compared to the Fumatech ionomer, since the perfluorinated polymer membranes contained well-connected hydrophilic ionic channels. Furthermore, the Aquivion-TMA membrane exhibited an excellent flow electrode capacitive deionization performance and stability because of its low equivalent weight and its chemical structure containing short side-chains. Although the perfluorinated polymers demonstrated high alkaline anion conductivities, their alkaline stabilities were found to be poorer than that of the hydrocarbon-based Fumatech ionomer, owing to the strong electron-withdrawing characteristics of the perfluorinated structure. Consequently, perfluorinated polymer-based anion exchange membranes require further study to improve their chemical stabilities through modification of their chemical structures to ultimately expand their applications.

## Figures and Tables

**Table 1:** Physical properties of Nafion-TMA, Aquivion-TMA, and Fumatech.

Ionomer	IEC [meq/g]		Water Uptake (wt%)	Swelling Ratio (%)		Conductivity @ 25 °C (mS/cm)	CF (mS/cm)
	Theoretical	Titration		Area	Thickness		
Nafion-TMA	0.91	0.88	21	12	5	27	1.31
Aquivion-TMA	1.15	1.06	23	15	5	38	1.67
Fumatech	1.6-2.1	1.84	34	17	7	10	0.30
